# Injectable and Self-Invigorating Hydrogel Applications in Dentistry and Periodontal Regeneration: A Literature Review

**DOI:** 10.7759/cureus.29248

**Published:** 2022-09-16

**Authors:** Unnati Shirbhate, Pavan Bajaj

**Affiliations:** 1 Periodontics and Implantology, Sharad Pawar Dental College, Datta Meghe Institute of Medical Sciences, Wardha, IND; 2 Periodontics, Sharad Pawar Dental College, Datta Meghe Institute of Medical Sciences, Wardha, IND

**Keywords:** choindritin sulfate (cs), gel, poly-vinyl, ecm, polymer, ha, hydrogel, 3d bioprinting, col, chitosan

## Abstract

Hydrogels are thought of as unique polymers utilized to build new materials, and two key factors that impact their features are their hydrophilicity and the degree of cross-linking of the polymer chains. An injectable hydrogel is based on the hypothesis that certain biomaterials can be injected into the body as a liquid and progressively solidify there. The scientific research community was intrigued and interested by its discovery. The hydrophilic polymers that are used to make hydrogels can typically be split into two groups: natural polymers derived from tissues or other sources of natural materials, and synthetic polymers produced by combining principles from organic chemistry and molecular engineering. A variety of organic and synthetic biomaterials, such as chitosan, collagen or gelatin, alginate, hyaluronic acid, heparin, chondroitin sulfate, polyethylene glycol, and polyvinyl alcohol, are used to generate injectable hydrogels. A promising biomaterial for the therapeutic injection of cells and bioactive chemicals for tissue regeneration in both dentistry and medicine, injectable hydrogels have recently attracted attention. Since injectable scaffolds can be implanted with less invasive surgery, their application is seen as a viable strategy in the regeneration of craniofacial tissue. Treatment for periodontitis that effectively promotes periodontal regeneration involves injecting a hydrogel that contains medications with simultaneous anti-inflammatory and tissue-regenerating capabilities. The advantages of injectable hydrogel for tissue engineering are enhanced by the capability of three-dimensional encapsulation. A material's injectability can be attributed to a variety of mechanisms. The hydrogels work well to reduce inflammation and promote periodontal tissue regeneration.

## Introduction and background

Due to the chemical or physical cross-linking of individual polymer chains, a hydrogel is a three-dimensional (3D) network of hydrophilic polymers that can swell in water and store a lot of water while keeping the structure. Whichterler and Lim made the first discovery of hydrogels in 1960 [1]. The degree of polymer chain cross-linking and hydrophilicity are two key elements that affect the properties of hydrogels. The ability of hydrogels to retain water is due to the presence of functional groups such as hydroxylic (-OH), carboxylic (-COOH), amidic (-CONH-), primary amidic (-CONH_2_), and sulphonic (-SO_3_H) groups within the polymer network [2]. The hydrogel systems have been proposed as potential carriers or scaffolding for pharmaceuticals. They have also been thoroughly investigated for a number of biomedical applications due to their benefits, which include biocompatibility, permeability to oxygen and nutrients, physical qualities similar to those of the original extracellular matrix (ECM), and programmable physical and mechanical properties [3].

**Figure 1:**
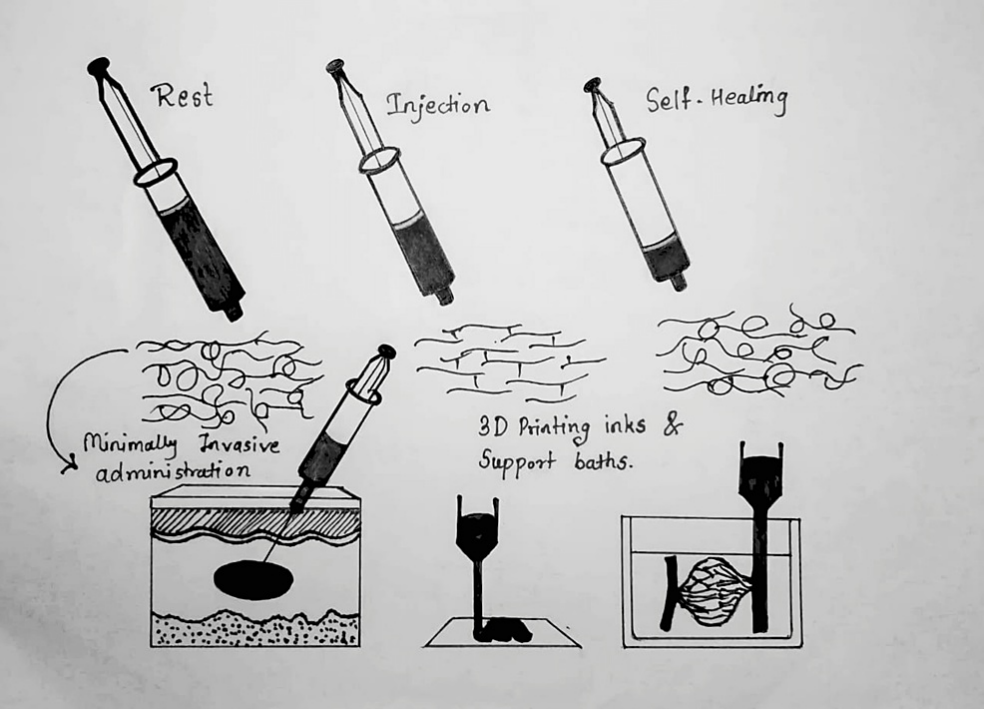
Shows a self-healing injectable hydrogel's behavior schematically At the first place, at rest, the material exhibits gel-like properties; in the second place, fluidization under shear as a result; and finally, self-healing of the original structure and mechanical characteristics after flow. Source: Ref. [5].

An injectable hydrogel is based on the concept that some biomaterials can be injected into the body as a liquid and then solidify in place. Injectable hydrogels have generated a lot of attention in the areas of medication delivery, tissue engineering, and dermal fillers because they have the necessary physicochemical qualities to be injected in situ into the body [4]. One especially intriguing family of self-healing hydrogels is one that may be injected or printed (in the context of 3D printing). As schematically shown in Figure 1, these self-healing injectable hydrogels are capable of momentarily fluidizing under shear stress and then regaining their original structure and mechanical characteristics after releasing the applied tension [5].

## Review

History of hydrogels

The first hydrogel poly-2-hydroxyethyl methacrylate (PHEMA) was created and described in 1960 by Whichterler and Lim. They used it to make moisture-absorbing contact lenses. Modern hydrogels are demonstrated and resembled by their 3D crosslinking structure. The scientific research community was intrigued and interested by its discovery. Then, in the 1980s, Lim and Sun created calcium-alginate gel composites for islet-droplet microcapsule cell embedding [6].

According to Buwalda et al., there have been three distinct generations of hydrogels. The first generation of hydrogels mostly consisted of gels with diverse crosslinking techniques created by chemically altering a monomer or polymer using an initiator. After this time, in the 1970s, the significance of hydrogels increased to a new level as stimuli-responsive properties were incorporated into the hydrogels, allowing second-generation hydrogels to react to a variety of highly specific stimuli, including changes in pH, temperature, or the concentration of certain biomolecules in a solution. The focus switched to the creation of stereo-complexed biomaterials and hydrogels joined through physical interactions in the third-generation hydrogels. These changes prompted scientists to focus their efforts more intently on creating the current "smart hydrogels," which can be tailored to acquire specific qualities like stimulus responsiveness and adjustable mechanical and other physicochemical properties [7].

Building blocks for the preparation of injectable hydrogels

A variety of materials are used to create injectable hydrogels. Generally speaking, there are two types of hydrophilic polymers that are utilized to make hydrogels: natural polymers taken from tissues or other natural sources, and synthetic polymers created utilizing organic chemistry and molecular engineering concepts. Building blocks of synthetic and biocompatible natural polymers are used to create the injectable hydrogels shown in Table 1 [7].

**Table 1:** Shows biocompatible natural polymer and synthetic polymer building blocks for the preparation of injectable hydrogels Source: Ref. [7].

Natural sources	Synthetic polymers
By using covalent or physical crosslinking (such as ionic or hydrogen bonding) to create injectable hydrogels, these organic polymers have been used as building blocks (e.g., reaction of functional groups on modified polymers).	Synthetic polymers are employed in conjunction with natural polymers or biomimetic peptides to promote cell adhesion, migration, and protein release because they lack the intrinsic biochemical cues for contact with cells.
Hyaluronic acid	Polyethylene glycol (PEG)
Chitosan	Polyvinyl alcohol (PVA)
Heparin	Poly N-isopropylacrylamide (PNIPAAm)
Alginate	Polycaprolactone (PCL)
Fibrin	
Collagen	
Chondroitin sulfate	
Silk	

Types of injectable hydrogels

Natural and synthetic biomaterials, such as chitosan, collagen or gelatin, alginate, hyaluronic acid, heparin, chondroitin sulfate, polyethylene glycol (PEG), and polyvinyl alcohol (PVA), are used to form injectable hydrogels. Chemical techniques that use covalent crosslinking produced by enzymes, physical techniques that use weak secondary forces, chemical techniques that use photo-cross-linking, physical techniques that use Michael addition, and chemical techniques that use click chemistry can all be used to create injectable hydrogels that are ion-sensitive, pH-sensitive, or temperature-sensitive [8].

Chitosan-Based Hydrogels

Chitosan is a cationic polymer consisting of glucosamine and N-acetylglucosamine that is found naturally. Because of its biocompatibility and biodegradability, chitosan is frequently used in the pharmaceutical and medical fields. Because of the many amino groups on its backbone, chitosan is a great choice for creating injectable self-healing hydrogels based on imine linkages [9].

Phosphate-Based Hydrogels

A fresh, injectable oligo-polyethylene-glycol fumarate (OPF) gel for bone healing with continuous release of phosphate ions, improved electrical conductivity, and mechanical strength. The mechanical stability and electrical conductivity of gel formulations were shown to be improved by a carbon nanotube nanocomposite, and the inclusion of two-dimensional (2D) black phosphorus nanosheets allowed a continuous release of phosphate ions by environmental oxidation [10].

Alginate-Based Hydrogels

Alginate hydrogels with biomedical uses fall into two categories: "Physical" or "reversible" gels are held together by molecular entanglements, hydrophobic forces, and ionic or hydrogen bonding, as opposed to "chemical" or "permanent" gels, which are produced when stable covalent connections crosslink networks. Alginate hydrogels are excellent alternatives for drug carriers because of their high water content, nontoxicity, soft consistency, biocompatibility, and biodegradability. They can transport low-molecular-weight drugs as well as macromolecules like proteins and DNA either sustainably or locally [11].

Hyaluronic Acid (HA)-Based Hydrogels

Because it may imitate tissue ECM and has the ability to influence cell behavior during tissue regeneration, such as cartilage and pulp regeneration, HA has been widely used to make injectable hydrogels. Since HA's molecular structure contains a variety of primary and secondary hydroxyl and carboxyl groups, a bifunctional small-molecule crosslinking agent can also be utilized for this purpose. This results in a HA hydrogel with the optimum composition, shape, hardness, and biological activity [12].

Collagen (col)- or Gelatin (gel)-Based Hydrogels

Due to their high levels of biocompatibility, biodegradability, bioactivity, and diversity, gelatin methacryloyl (GelMA) hydrogels are frequently employed for tissue healing. By adding double bonds to the gelatin polymer chains, which under photoinitiation quickly form hydrogels, GelMA hydrogel is created. Lithium acylphosphinate salt (LAP), a blue light initiator, facilitates preparation and speeds up the gelation process. It poses no threat. Injectable hydrogel made from GelMA hydrogel works well and may be molded using 3D printing [13].

Fibrin-Based Hydrogels

Known as a helpful cell-transplantation matrix, they can improve cell adhesion, proliferation, differentiation, and migration in a 3D scaffold. Fibrin is a naturally occurring fibrous protein implicated in blood clotting. Scaffolds have been constructed using fibrin, either by itself or in conjunction with other substances, for applications involving cartilage tissue engineering. A unique injectable hydrogel system has been created using PEG, stem cells generated from human amniotic fluid, and fibrin-based hydrogels that can promote in situ neovascularization and cause a fibrin-driven angiogenic host response [14].

Elastin-Based Hydrogels

For numerous biological applications, injectable hydrogels produced from ECM proteins like elastin hold considerable promise. The primary problems of these hydrogels are their lack of mechanical strength, use of cytotoxic chemicals, and fixed gelling behavior [15].

Chondroitin Sulfate (CS)-Based Hydrogels

The safety and high biocompatibility of CS, a common material for cartilage tissue engineering scaffolds that exhibits quick gelation, outstanding mechanical capabilities, and delayed degradation qualities, have been thoroughly explored and documented [16].

PEG-Based Hydrogels

PEG-based hydrogels and their derivatives have received a lot of attention recently due to their capacity to be well tolerated in vivo in the context of drug administration and tissue engineering applications. Because they may be administered easily and painlessly by injecting low-viscosity precursor polymer solutions, injectable, in situ gelling counterparts improve the potential applications of these hydrogels [17].

PVA-Based Hydrogels

For the in situ production of hydrogels under physiological conditions, new PVA compounds with a variety of pendant chemoselective characteristics have been developed. For the first time, PVA was modified by adding thiol, cysteine 1,2-aminothiol, and aminooxy side chains by direct carbamate connections from protected nucleophilic functionalities to the hydroxyl groups of PVA [18].

Shape Memory (SM) and Self-Healing (SH) Hydrogels

Both SM and SH have their roots in reversible interactions. It is quite difficult to synthesize hydrogels using both SH and SM [19].

Interpenetrating Polymer Network (IPN) Hydrogels

To address the conundrum of choosing between a complex structure and an easy biodegradability, a novel method for creating biodegradable hydrogels with an IPN structure that consists of peptide self-assembling networks and a covalently cross-linked network has been proposed [20].

Double Network (DN) Hydrogels

Due to their outstanding mechanical and chemical adaptability, DN hydrogels have become leading contenders for tissue engineering. DN hydrogel formulations combined with processing advancements (such as additive manufacturing and injection) have produced a remarkable set of findings that significantly advance the development of systems that can address the complex environment around tissues and allow for individualized fabrication techniques [21].

Programmable Hydrogels

Programmable hydrogels are hydrogels that have the ability to periodically, reversibly, and sequentially alter their properties and functionalities. Using the aforementioned concepts, programmable hydrogels that are induced to undergo functional changes could be produced for a range of intriguing applications [22].

3D Printed Hydrogels

In tissue engineering and regenerative medicine, 3D bioprinting, one of the most recent biotechnologies, is frequently used to produce complex artificial organ and tissue designs that closely resemble genuine organs and tissues. In order to restore functional and site-specific tissues or organs, bioprinting is the additive deposition of cell-loaded hydrogels in a specified structural framework [23].

Injectability mechanisms of injectable hydrogels

A material's injectability can be attributed to a variety of mechanisms. These mechanisms are divided into three major categories: The first mechanism is in situ gelling liquids - solutions or liquids that normally flow but harden into gels when injected into the body. The second mechanism is injectable gels - even though certain gels are created ex vivo, their shear thinning qualities and capacity to restore their hydrogel shape following relaxing procedures make them suitable for injection. The third mechanism is injectable particles - in addition to the two types of injectable systems previously discussed, the third class of injectable particles also has the ability to be injected when immersed in a liquid phase. Depending on the desired outcomes, particle sizes might be nano-, micro-, or macroscale [24].

Applications of injectable hydrogels

Therapeutic Applications in Dentistry

A promising biomaterial for the therapeutic administration of cells and bioactive compounds for tissue regeneration in dentistry and medicine is injectable hydrogels. They are suitable for minimally invasive surgical operations in a clinical context because they offer adaptable tissue-like qualities, regulated degradation and release behavior, and the ability to conform to the 3D defect upon gelling. A recent development in tissue engineering used a biomaterial scaffold and bioactive substances to encourage endogenous cell migration and tissue repair, further demonstrating the potential of the "homing" strategy for dentin-pulp and craniofacial regeneration [25]. Injectable biomaterials are regarded as great candidates for pulp and dentin regeneration because of the tooth root canal's small size and uneven shape. The first organic biomaterial employed as an injectable gel for pulp regeneration was collagen. As a dental stem cell transporter, fibrin was treated with PEG to create a poly-ethylene-Gylated fibrin hydrogel with a slower rate of breakdown [26]. By modifying its physical and chemical characteristics, the hydrogel's shelf life may be increased to three years, and it can be successfully utilized as the only maxillofacial material. As a denture adhesive, hydrogels have a number of advantageous characteristics [27]. Since injectable scaffolds can be inserted with less invasive surgery, lowering the risk of surgical problems and enhancing postoperative recovery, their usage is seen as a potential strategy for the regeneration of craniofacial tissue [28]. Laden HA injectable hydrogels as a biomaterial for the encapsulation of human dental pulp cells demonstrate significant clinical potential for endodontic regeneration therapy. In order to regenerate bone tissue, CS-based hydrogels can greatly increase cell proliferation and cell adhesion [29].

Therapeutic Applications in Periodontal Regeneration

Treatment for periodontitis that effectively promotes periodontal regeneration involves injecting a hydrogel that contains medications with simultaneous anti-inflammatory and tissue-regenerating capabilities. The hydrogels showed outstanding self-healing abilities, an expedient gelation process, and injectability [30]. To reduce inflammation and encourage tissue regeneration, local medication delivery has been used as a successful method. The CS-based delivery approach can be depended upon to deliver the encapsulated active drugs to the disease site within periodontal pockets by utilizing injectable chitosan hydrogels with modulable physico-chemical characteristics [29]. Emdogain is based on a derivative of porcine enamel matrix, a combination of proteins supplied in an aqueous gel solution made of propylene glycol alginate and including amelogenin (90%) along with a few other nanomelogenin such as ameloblastin, enamelin, and tuftelin. Emdogain has been shown to regenerate a variety of periodontal tissues, including connective tissues like the periodontal ligament as well as osseo-like tissues, acellular cementum, and alveolar bone [31]. Aspirin/erythropoietin was used to fill the successfully created and proven to be helpful in periodontium regeneration CS/gelatin hydrogel [32]. The dual drug-loaded oxidized dextran and phenylboronic acid-functionalized polyethylene imine hydrogel, a novel potential therapeutic agent, may be beneficial for the therapy of chronic periodontitis with diabetes mellitus [33]. Innovative hybrid hydrogel offers enormous potential as an injectable platform technology with a variety of applications in the eradication of mouth infections such as periodontal disease and pulpal pathology [34].

Injectable Hydrogel Delivery for Tissue Engineering

The use of injectable hydrogels as a delivery system for topical and localized medication delivery appears promising. The advantages of injectable hydrogel for tissue engineering are enhanced by the capability of 3D encapsulation. An injectable hydrogel must strike a compromise between mechanical properties, carrier capacity, and processability [35]. It has been demonstrated that among the several scaffolds for bone tissue engineering applications, hydrogels are attractive templates for bone regeneration due to their similarity to the natural ECM. Chitosan, a natural biopolymer, has attracted a lot of attention since it can be utilized to produce thermo/pH-responsive injectable hydrogels [36].

Future directions and current status

Due to their adaptability, injectable hydrogels, a subset of hydrogel, have drawn significant interest in biological applications. According to reports, the injectable hydrogel can be used in a variety of biomedical treatments, such as tissue engineering for cartilage and bone as well as periodontal implants and submucosal fluid cushions. In addition to being simple to implant, this kind of hydrogel can be customized to fit certain purposes [37].

## Conclusions

Due to its potential for less invasive local drug administration, more precise implantation, and site-specific drug delivery into difficult-to-reach tissue regions and into interface tissues, where wound healing takes time, injectable hydrogels have attracted a lot of interest in the biomedical industry. It is still extremely desirable to create an injectable hydrogel with self-healing capabilities for continuous, controlled drug delivery. For prolonged protein release, targeted drug delivery, and tissue engineering, hydrogels, microgels, and nanogels have become efficient and useful platforms because of their high biocompatibility and microporous structure with adjustable porosity in periodontal regeneration. Among other things, the hydrogels are successful in reducing inflammation and promoting periodontal regeneration.
